# Two novel fish paralogs provide insights into the Rid family of imine deaminases active in pre-empting enamine/imine metabolic damage

**DOI:** 10.1038/s41598-020-66663-w

**Published:** 2020-06-23

**Authors:** Stefania Digiovanni, Cristina Visentin, Genny Degani, Alberto Barbiroli, Matteo Chiara, Luca Regazzoni, Flavio Di Pisa, Andrew J. Borchert, Diana M. Downs, Stefano Ricagno, Maria Antonietta Vanoni, Laura Popolo

**Affiliations:** 1 Department of Biosciences, University of Milan, Milan, Italy; 2 Department of Food, Environmental and Nutritional Sciences, University of Milan, Milan, Italy; 3 Department of Pharmaceutical Sciences, University of Milan, Milan, Italy; 4 Department of Microbiology, University of Georgia, Athens, GA United States; 5 Present Address: Department of Chemical Biology I, University of Groningen, Groningen, The Netherlands; 6 Present Address: National Bioenergy Center, National Renewable Energy Laboratory, Golden, CO United States

**Keywords:** Biochemistry, Structural biology, Enzymes, Metabolic pathways

## Abstract

Reactive Intermediate Deaminase (Rid) protein superfamily includes eight families among which the RidA is conserved in all domains of life. RidA proteins accelerate the deamination of the reactive 2-aminoacrylate (2AA), an enamine produced by some pyridoxal phosphate (PLP)-dependent enzymes. 2AA accumulation inhibits target enzymes with a detrimental impact on fitness. As a consequence of whole genome duplication, teleost fish have two *ridA* paralogs, while other extant vertebrates contain a single-copy gene. We investigated the biochemical properties of the products of two paralogs, identified in *Salmo salar*. _Ss_RidA-1 and _Ss_RidA-2 complemented the growth defect of a *Salmonella enterica ridA* mutant, an *in vivo* model of 2AA stress. *In vitro*, both proteins hydrolyzed 2-imino acids (IA) to keto-acids and ammonia. _Ss_RidA-1 was active on IA derived from nonpolar amino acids and poorly active or inactive on IA derived from other amino acids tested. In contrast, _Ss_RidA-2 had a generally low catalytic efficiency, but showed a relatively higher activity with IA derived from L-Glu and aromatic amino acids. The crystal structures of _Ss_RidA-1 and _Ss_RidA-2 provided hints of the remarkably different conformational stability and substrate specificity. Overall, _Ss_RidA-1 is similar to the mammalian orthologs whereas _Ss_RidA-2 displays unique properties likely generated by functional specialization of a duplicated ancestral gene.

## Introduction

Metabolism is composed of different arrangements of enzyme-catalyzed reactions that convert a precursor into a product through a series of metabolic intermediates. Among hundreds of metabolites generated from catabolic and anabolic reactions, small molecules endowed with high reactivity and toxicity may arise and cause metabolite damage^1^. Cells protect their components using two strategies primarily aimed to (*i*) repair anomalous metabolites and reconvert them to the original form^2–4^, or (*ii*) pre-empt damage by converting the harmful compound into a benign one^2^. A demonstration of the biological relevance of this phenomenon is the increasing number of genes with previously unknown functions that encode enzymes involved in metabolite damage control across all living organisms^1,2,5^. A notable example is the Rid superfamily, known for many years as the YjgF/YER057c/UK114 family, for which a common *in vitro* activity as imine deaminase and involvement in a pre-empting mechanism has been elegantly demonstrated^1,6,7^. The term Rid (reactive intermediate deaminase) refers to the hydrolytic deamination of highly reactive and labile enamines/imines to α-ketoacid and ammonia, a reaction that would be otherwise relatively slow if left to occur spontaneously^6,7^.

The Rid superfamily is composed of 8 families: RidA is present in all domains of life whereas Rid1 to Rid7 families are unique to prokaryotes and found predominantly in bacteria^8,9^. From the Rid1 to Rid3 families, a conserved arginine residue (Arg105) is believed to be sufficient to predict the imine hydrolyzing activity of the proteins and this residue is also shared by all RidA enzymes from *Eukarya* (Arg107). While Rid proteins from different families are normally observed in prokaryotes^8,9^, for still unclear reasons eukaryotes restricted their repertoire only to members of the RidA family. In most eukaryotes, RidA is a cytosolic enzyme encoded by a single-copy gene. However, a few notable exceptions have been described. For example, the yeast *Saccharomyces cerevisiae*, beside the canonical RidA (Hmf1p/Yil057cp), displays the presence of another paralogous protein (Mmf1p/Yil051cp) that is imported into the mitochondrion^10–12^. In plants (maize and *Arabidopsis thaliana*), the RidA enzyme is localized in the chloroplast^13^. Interestingly, all teleosts (bony fish) have two RidA paralogs, most probably as a consequence of Clade-specific whole genome duplication (WGD), but neither of these paralogs has been characterized so far.

The best-characterized function of RidA is the quenching of specific enamines. The most physiologically relevant compounds are 2-aminoacrylate (2AA), which is an obligate reaction product of the pyridoxal-5′-phosphate (PLP)-dependent serine dehydratases and cysteine desulfhydrases [see^1^ for a review] and 2-aminocrotonate (2AC), the product of threonine dehydratase. The enamines 2AA and 2AC are believed to readily tautomerize to 2-iminopropionate (2-iminopyruvate) and 2-iminobutyrate, respectively. RidA accelerates the conversion of these compounds to the final products pyruvate and α-ketobutyrate, respectively, and ammonia. The conversion to benign products catalyzed by RidA enzymes constitutes a new paradigm in metabolite damage control^1^.

When accumulated, 2AA can form adducts with PLP or with active site residues of PLP-dependent target enzymes, but also with yet unknown targets that are irreversibly inactivated^7,14,15^. Alanine racemase^14^, serine hydroxymethyltransferase^16^, branched chain amino acid transaminase B^7^ and aspartate aminotransferase transaminase^17^ are among the physiologically relevant targets identified so far^18^. A wide transcriptomics and metabolomics integrated study identified folate and branched amino acid metabolism as the most perturbed pathways in a *Salmonella enterica ridA* null mutant^19^. In general, elimination of RidA activity in the cell reduces its fitness and causes pleiotropic phenotypes^7,20^. An outstanding example is the different consequence caused by the lack of RidA for different organisms. In *S. enterica* loss of RidA causes serine and cysteine sensitivities, pyruvate excretion, and a motility defect^15,21–23^. Deletion of *ridA* from *Campylobacter jejuni* causes defects in motility, autoagglutination and in phage infectivity^24^. Yeast cells lacking the mitochondrial RidA (Mmf1p) exhibit a severe defect in mitochondrial genome stability due to 2AA stress generated by the mitochondrial serine dehydratase inside the organelle^12^. In plants (maize and *A. thaliana*) RidA ablation leads to failure in root growth^13^.

While classification of Rid families on the basis of amino acid sequence conservation is well established, the *in vitro* activity and the specificity of eukaryotic RidA proteins are still poorly characterized^8,9^. In addition to bioinformatic analyses, assays of deaminase activity on a wide spectrum of substrates, *in vitro* reconstitution of reactions of the intermediate metabolism, and also *in vivo* complementation tests are crucial to define the biochemical features and the biological role of a RidA protein^1^.

In the frame of a broader study on eukaryotic RidA, we previously characterized the biochemical properties of goat UK114 (_Ch_RidA), an acid-soluble homotrimeric liver protein that became the object of intense studies more than twenty years ago due to its peculiar immunogenic properties, well before the proof of its biochemical activity was obtained^25^. Here, we identified _Ch_RidA homologs in teleosts (bony fish), a class of vertebrates in which, to the best of our knowledge, the Rid proteins have never been studied. Teleosts represent an interesting case of study for the biochemical characterization of novel RidA proteins since, as a consequence of a relatively recent teleost-specific WGD^26–28^, most of the currently available teleost genomes carry two paralogous copies of several genes. *Salmo salar* (Atlantic salmon), a teleost, is one of the most studied fish species due to its significant economic and ecological value. A nearly complete reference assembly of the genome has been recently released^29^ and is publicly accessible through a dedicated web portal, Salmobase 2.0^30^. The availability of a high-quality genomic assembly offers the unprecedented opportunity to study the mechanisms that shape the genomic and chromosomal reorganization in vertebrates following a WGD^31^.

In the present study, we identified two functionally active RidA genes in the salmon genome, here named *ridA-1* an*d ridA-2*, and we performed an extensive biochemical and structural characterization of their products (_Ss_RidA-1 and _Ss_RidA-2). Both proteins exhibited *in vitro* imine deaminase activity, but markedly different substrate specificity. The determination of the crystallographic structures of _Ss_RidA-1 and _Ss_RidA-2 revealed fine differences between the isozymes that helped understanding the differences in substrate specificity and in protein stability between _Ss_RidA-1 and _Ss_RidA-2. Remarkably, both isozymes could functionally replace *in vivo* a bacterial RidA protein in relieving 2AA stress.

## Results

### Identification of two functionally active RidA-encoding genes in *Salmo salar*

To detect RidA homologs in *S. salar*, we used a sequence similarity search based on BLASTP and a comparative genomics analysis. By BLASTP, five sequences with similarity to the human RidA protein (_Hs_RidA, 136 residues) were detected. Three of these sequences were ascribable to a single 137 residues-long protein, here named _Ss_RidA-1, sharing 71% identity with _Hs_RidA. The first sequence predicted from a *S. salar* genome assembly (NW_012336577.1) was identical to a second one deduced from cDNA (head kidney, BT056740.1/ACM08612.1) except for a Gly19Glu replacement caused by the conversion of the GGG codon for glycine into the GAG codon for glutamate, likely originated from a point mutation or a sequencing error being Gly19 highly conserved in RidA proteins. _Ss_RidA-1 matched the *ridA-1* locus on *S. salar* chromosome 10 (Chr ssa10). A third sequence derived from a thymus cDNA (BT0557768.1/ACM09640.1) and was a chimeric _Ss_RidA-1 protein originated from the retention of part of the fourth intron of the *ridA-1* gene. Since this sequence likely derives from an incompletely processed _Ss_RidA-1 transcript, no further studies were performed on it.

Two additional sequences were ascribable to a putative protein of 135 residues, hereafter referred to as _Ss_RidA-2, sharing 61% identity with _Hs_RidA. Both sequences derived from cDNA libraries obtained from pyloric caeca [BT047927.2^32^] and from mixed brain, kidney and spleen tissues (BT058640.1). This second RidA homolog matched to a locus on chromosome 14 (Chr ssa 14) indicating that it was the product of a single gene here named *ridA-2*.

**Figure 1:**
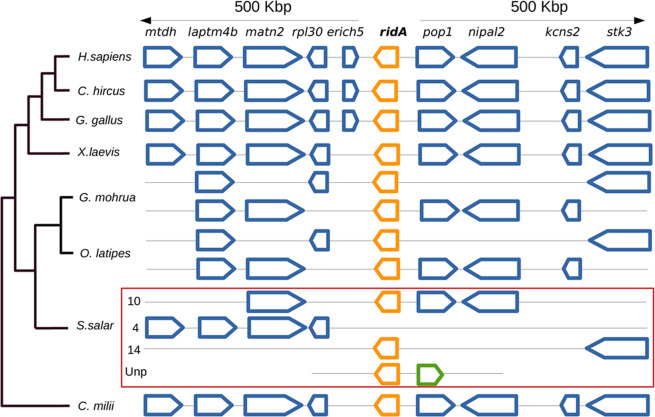
Schematic representation of gene order and orientation of a genomic locus of 1 Mbp centered on the *ridA* gene (in bold) in a selection of vertebrate genomes. Gene names are indicated on the top. Genes are represented by thick arrows directed in the sense of transcription. Size is proportional to the size of the gene body. *ridA* gene is represented in orange. Conserved flanking genes are colored blue. A *ridA* flanking gene that is not conserved in at least 3 distinct species is shown in green. For *Salmo salar*, only chromosome numbers are reported next to the scheme of each paralogous *ridA* locus. Unp: unplaced scaffold. Phylogenetic relationships between species are represented by a simple cladogram. Species are indicated by their scientific names: *Homo sapiens*, *Capra hircus* (goat)*, Gallus gallus* (domestic chicken), *Xenopus laevis* (frog)*, Gadus mohrua* (cod), *Orizias latipes* (Japanese rice fish), *Salmo salar* (Atlantic salmon) and *Callorhincus milii* (elephant shark).

In addition, a synteny-based approach was used to infer the organization of the *ridA* locus in a carefully selected collection of publicly available vertebrate genomes. A 1 MBp-chromosome region centered on the human *ridA* gene (for simplicity here indicated in lowercase) was analyzed. A remarkable conservation among *ridA* loci was observed (Fig. 1). With the only exception of *erich5*, absent in the African clawed frog *Xenopus laevis* and in the elephant shark *Callorhincus milii* (a cartilaginous fish), the genes surrounding human *ridA* showed a striking conservation in order and orientation within all the lineages having a single-copy *ridA* gene (Fig. 1). Consistent with the teleost-specific WGD at the basal divergence from the Osteichthyan fish class [~320 million years ago (Mya)]^26–28^, two copies of *ridA* were observed in all bony fish genomes included in our analyses. Representative loci shown in Fig. 1 are from *Gadus mohrua* (cod) and *Orizia latipes* (Japanese rice fish). Both loci showed only a partial conservation of gene content and order, most likely due to differential gene loss (Fig. 1).

An additional WGD event has been described in the common ancestor of the salmonid lineage, ~80 Mya after their divergence from the Esociformes fish order, and this justifies the presence of even four copies of a given gene in the salmonid species^29^. Interestingly, and consistently with the additional WGD event, four *ridA* loci were detected in the genome of *Salmo salar*. The locus present on chromosome 4 (Chr ssa04) conserved the typical order of the upstream genes but lacked all the downstream genes including *ridA* itself, suggesting that it was a remnant of extensive chromosomal rearrangements. By contrast, the other three loci contained a *ridA* gene. The *ridA* locus present on Chr ssa10 retained the human homologs *matn2*, *pop1* and *nipal2* around the *ridA-1* gene, encompassing 7,674 bp and coding for _Ss_RidA-1. The *ridA* gene on Chr ssa14, encompassing 11,465 bp, corresponded to *ridA-2*, and the associated genomic locus showed the lack of all the flanking genes except *stk3*. Both salmon *ridA* genes share an identical gene structure consisting of 6 exons and both genes are transcribed since full cDNA sequences were annotated as mentioned above. The third *ridA*-like gene resides in an unplaced scaffold (Unp) of 7,628 bp (NW_01235881), and has a single exon encoding a truncated RidA of 125 aminoacyl residues sharing only 41.47% identity with _Hs_RidA protein. This anomalous sequence likely corresponds to an inactive retro-pseudogene since a corresponding cDNA sequence is not present in the database. Due to its anomalous features, it was excluded from subsequent analyses. Considerations regarding the relatively high variability in gene order and content compared to the archetypal human *ridA* locus are consistent with evolution by recombination and differential gene loss of these loci.

In conclusion, our BLASTP and comparative genomic analyses indicate that *S. salar* has two functionally intact paralogs, *ridA-1* and *ridA-2*, residing in different chromosomes, namely Chr ssa10 (Gene ID: 106561137) and Chr ssa14 (Gene ID: 100195988). The two paralogous proteins, _Ss_RidA-1 and _Ss_RidA-2, share 77% amino acid identity and were the object of further studies.

### Primary structure, expression and purification of _Ss_RidA-1 and _Ss_RidA-2

**Figure 2:**
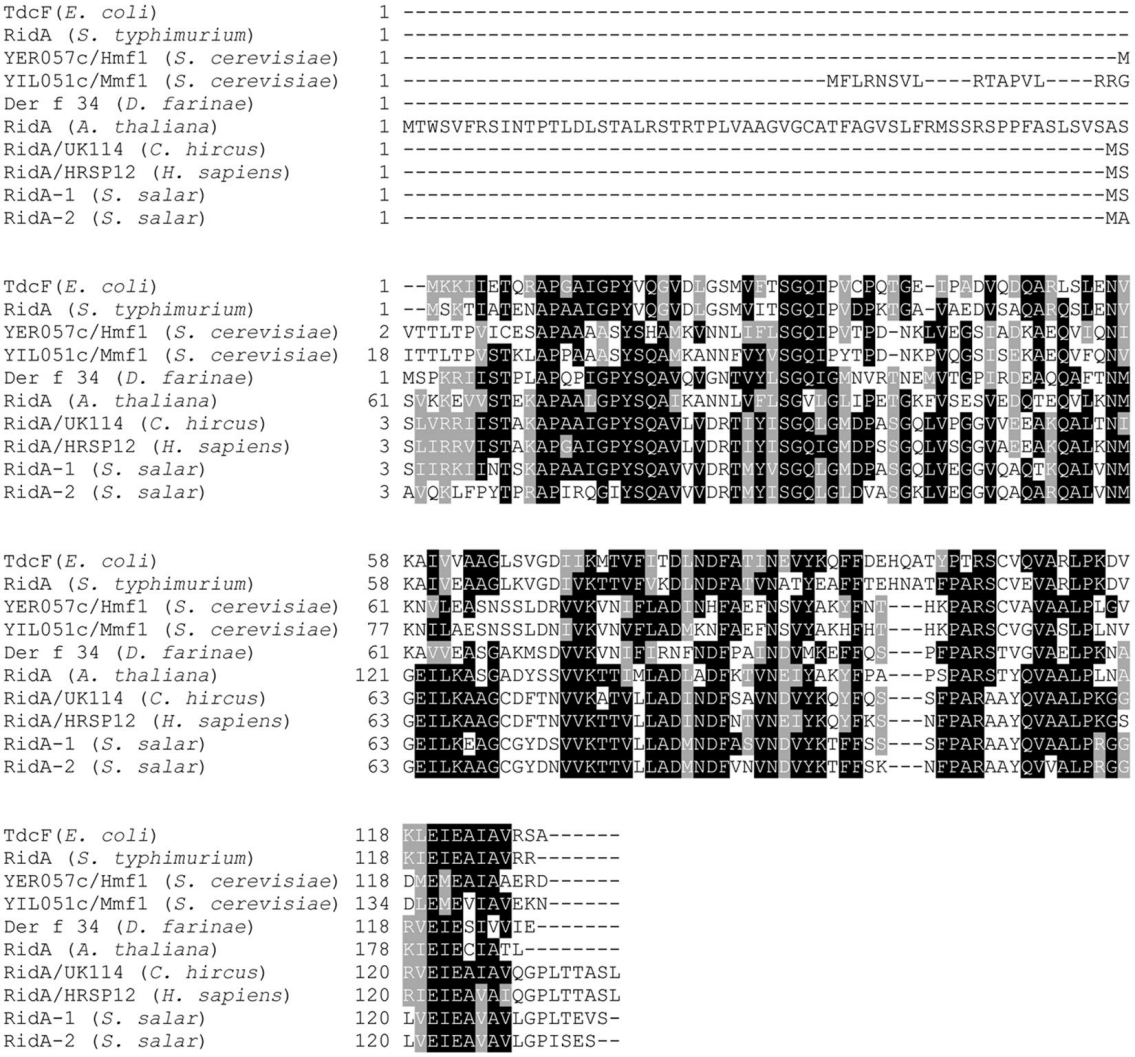
Salmon _Ss_RidA-1 and _Ss_RidA-2 proteins share conserved blocks of sequence motifs with prokaryotic and eukaryotic orthologous proteins. (A) Multiple alignment of the indicated RidA protein sequences using Clustal Omega and BOXSHADE default parameters. The aminoacyl sequences aligned in the figure are: *Escherichia coli* TdcF (P0AGL2); *Salmonella typhimurium* RidA (Q7CP78); *Saccharomyces cerevisiae* YER057c/*HMF1* (P40037) and YIL051c/*MMF1* (P40185); *Dermatophagoides farinae* (house dust mite) Der f 34 (A0A1J1DL12); *Arabidopsis thaliana* RidA (Q94JQ4); *Capra hircus* (goat) RidA/UK114 (P80601); *Homo sapiens* RidA (P52758); *Salmo salar* RidA-1 (A0A1S3KNQ3) and RidA-2 (C0H8I4). Black boxes indicate identical residues; grey boxes indicate similar residues. The import signals of Mmf1p and *A.thaliana* RidA are shown at the N-terminal end.

By multiple sequence alignment, several blocks of conserved aminoacyl residues were detected among bacteria, salmon and other eukaryotic RidAs of different origin (yeasts, insects, plants and mammals), which were experimentally demonstrated to be catalytically active (Fig. 2**)**. The frequency plot calculated from the multiple alignment using WebLogo^33^ showed the presence of several invariable residues in addition to the conserved R107, corresponding to R105 in bacteria (Fig. S1). The conserved residues are: A14, P15, Y21, S34, G35, N61, K78, F89, N93, F100, P105, R107, V112, L115, P116, E122 and E124. However, their role has yet to be defined.

To characterize the salmon RidA proteins, the CDS of _Ss_RidA-1 and _Ss_RidA-2 were cloned into pET-15b downstream to DNA regions encoding a 6xHis tag and a thrombin cleavage site. *E. coli* Rosetta (DE3) cells were transformed with the recombinant plasmids (see Table S1), and the proteins were expressed and purified as described under Methods. From LC/MS analyses, the purified _Ss_RidA-1 and _Ss_RidA-2 proteins were greater than 95% homogenous and had a monomeric molecular mass within 5 ppm from the expected values. Trypsin cleavage followed by MS fingerprinting resulted in peptide matching with the theoretical sequences for a total coverage greater than 95% for both _Ss_RidA-1 and _Ss_RidA-2 (data not shown).

### _Ss_RidA-1 and _Ss_RidA-2 exhibit *in vitro* deiminase activity, but different substrate specificity

**Figure 3:**
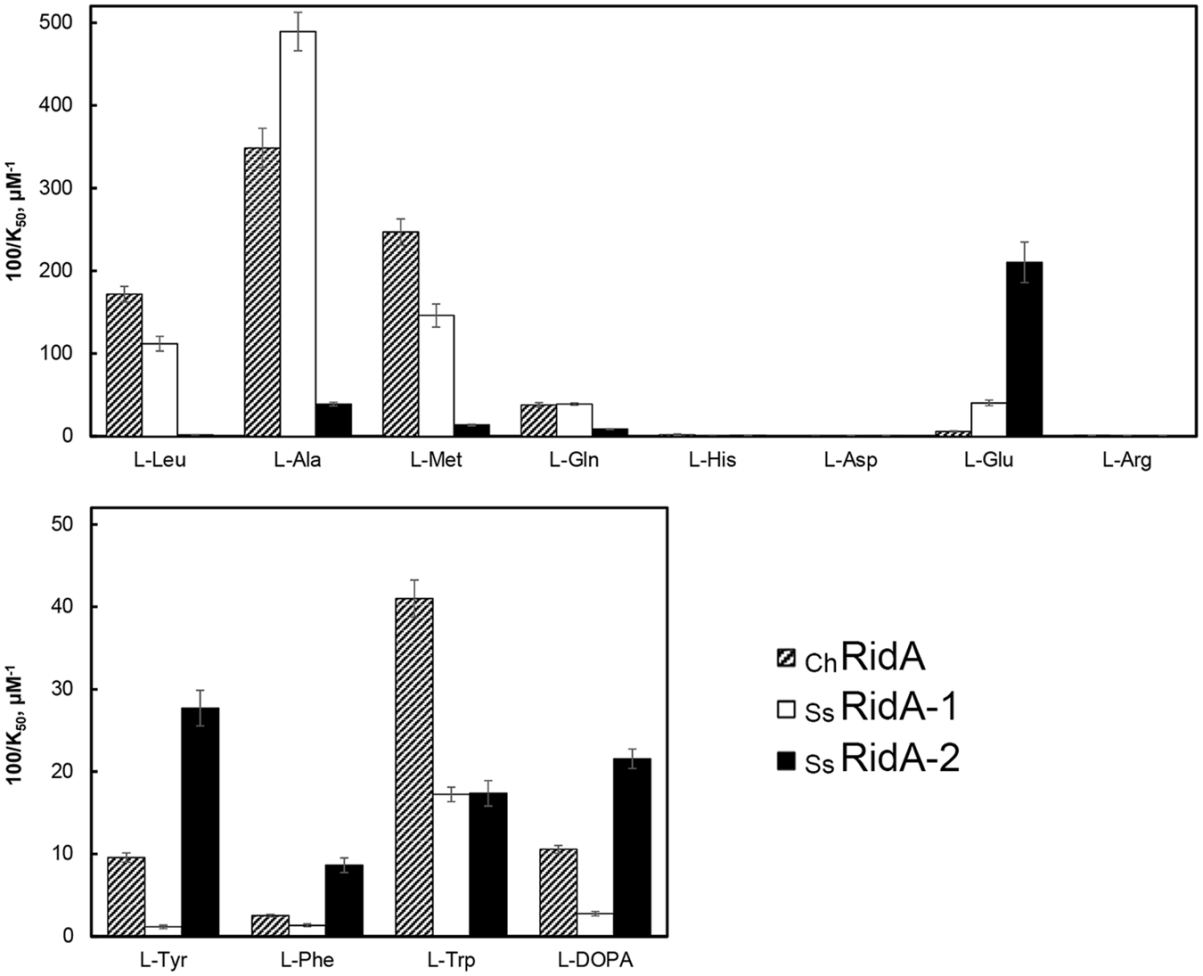
Deiminase activity and substrate specificity of salmon _Ss_RidA-1 and _Ss_RidA-2. The assays of RidA activity were carried out with purified _Ss_RidA-1, _Ss_RidA-2 and _Ch_RidA, which was used as the reference enzyme. The histograms show the values of 100/K_50_ for each enzyme, obtained by fitting three or more measurements, with the corresponding standard deviations (bars). The decrease of the rate of semicarbazone formation was monitored as an index of accelerated conversion of the *in situ* produced imino acid, from the indicated amino acids, to the corresponding α-keto acid and ammonia. The 100/K_50_ values are directly related to the catalytic efficiency of each RidA with the given imino acid, as described under Methods. The 100/K_50_ values and standard deviations are also reported in Table S2.

The activity of RidA enzymes was tested using FAD-dependent L-amino acid oxidases that can generate 2-imino acids (IA) upon oxidation of the corresponding L-amino acids^8,9,25^. The activity of _Ss_RidA-1 and _Ss_RidA-2 on a broad spectrum of IA was compared to that of goat RidA (_Ch_RidA), used as a reference being the best-characterized animal RidA^25^. Both _Ss_RidA-1 and _Ss_RidA-2 were active as imine deaminases but striking differences in substrate specificity between the isozymes were observed as highlighted in Fig. 3, which summarizes data reported in Table S2 and Fig. S2. _Ss_RidA-1 appeared to be active, like the goat enzyme, on IA derived from L-Ala, L-Met, L-Leu and L-Gln whereas _Ss_RidA-2 was one to two orders of magnitude less active on these IA except for that derived from L-Ala, *i.e*. 2-iminopyruvate (Fig. 3, *upper panel*). The latter is the stable tautomer of 2AA, the common product of serine dehydratase and cysteine desulfhydrase reactions. All the RidA enzymes showed limited activity on IA derived from aromatic amino acids and also on L-dihydroxy-phenylalanine (L-DOPA), but _Ss_RidA-2 displayed a weak preference for all these substrates except for L-Trp on which the goat enzyme showed a slightly higher activity (Fig. 3, *lower panel*). With respect to charged amino acids, no RidAs exhibited any significant activity in the presence of IA derived from basic amino acids and also from L-Asp. Interestingly, _Ss_RidA-2 stood out revealing the highest level of activity for the IA derived from L-Glu (Fig. 3, Fig. S2 and Table S2). These results suggest that _Ss_RidA-1 has substrate specificity similar to that of _Ch_RidA whereas that of _Ss_RidA-2 is remarkably different.

### *S. salar* RidA orthologs deaminate 2-aminoacrylate *in vivo*

**Figure 4:**
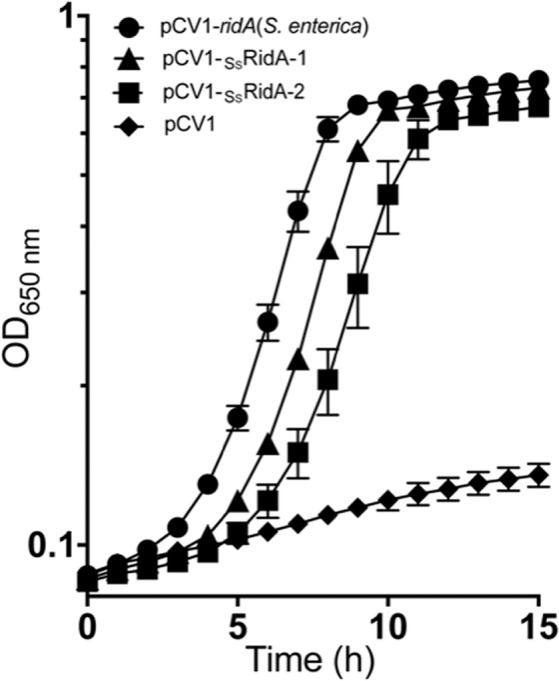
RidA orthologs from *Salmo salar* complement a *Salmonella enterica ridA* mutant. Strains were grown in minimal glycerol (20 mM) medium containing 5 mM serine with 0.2% (wt/vol) L-arabinose. Strains were *S. enterica ridA* mutant strains carrying the indicated plasmid, including vector only (pCV1), that expressed *S. enterica ridA* [pCV1-*ridA*(*S. enterica*)], *S. salar*_Ss_RidA-1 (pCV1-_Ss_RidA-1) or _Ss_RidA-2 (pCV1-_Ss_RidA-2). Error bars represent the standard error of the mean (SEM) from three biological replicates of each strain.

In *Salmonella enterica, ridA* mutants grown in minimal medium accumulate the reactive enamine 2-aminoacrylate (2AA), which is generated by the biosynthetic serine/threonine dehydratase (IlvA, EC:4.3.1.19) from the L-serine substrate^7^. In the presence of exogenous serine, 2AA damages a number of PLP-dependent enzymes, eliciting a severe growth defect^16,21^. To assess the ability of the two RidA orthologs from *S. salar* to deaminate enamines/imines *in vivo*, an *S. enterica ridA* mutant (DM14829) was transformed with plasmids pDM1616 or pDM1617 encoding _Ss_RidA-1 or _Ss_RidA-2, respectively (Table S1). The growth behavior of these strains, along with control strains harboring the vector only or expressing the *S. enterica ridA*, was assessed in minimal glycerol medium containing 5 mM L-serine (Fig. 4). As expected, the presence of pDM1439 (pCV1-*ridA*) restored full growth to the *S. enterica ridA* mutant, with or without induction of the *P*_*BAD*_ promoter by L-arabinose. In contrast, pDM1616 and pDM1617 allowed growth of the *S. enterica ridA* mutant, but only in the presence of exogenous L-arabinose. These data indicated that _Ss_RidA-1 and _Ss_RidA-2 have 2AA deaminase activity *in vivo*. The requirement for induction suggested that these proteins were less efficient 2AA deaminases than the *S. enterica* RidA enzyme. It is also possible that expression of the *S. salar* proteins was not as robust as that of *S. enterica* RidA from the pCV1 vector. However, since the synthetic genes were codon optimized for expression in *S. enterica*, this is considered unlikely. Protein stability may also account for the discrepancy between complementation constructs. Finally, there was a minor difference between _Ss_RidA-1 and _Ss_RidA-2, with expression of _Ss_RidA-1 allowing slightly better growth of the *S. enterica ridA* mutant than _Ss_RidA-2. These data suggest that 2AA could be a better substrate for _Ss_RidA-1 than _Ss_RidA-2, a hypothesis consistent with the observation that _Ss_RidA-1 exhibited a higher 100/K_50_ than _Ss_RidA-2 (Fig. 3) for the deamination of 2-iminopyruvate, the tautomer of 2AA directly generated by LAAO from L-Ala. The use of 2AA in *in vitro* assays is not feasible due to the labile nature of this compound.

### _Ss_RidA-1 and _Ss_RidA-2 have different conformational stability

The secondary structures of _Ss_RidA-1 and _Ss_RidA-2 were investigated by comparing their far-UV circular dichroism (CD) spectra with that of _Ch_RidA. As shown in Fig. S3, the three spectra were almost superimposable. Deconvolution of the spectra indicates that the three RidA proteins share a similar secondary structure content, in which α-helix and β-sheet account approximately for 18% and 28%, respectively (Table S3).

**Figure 5:**
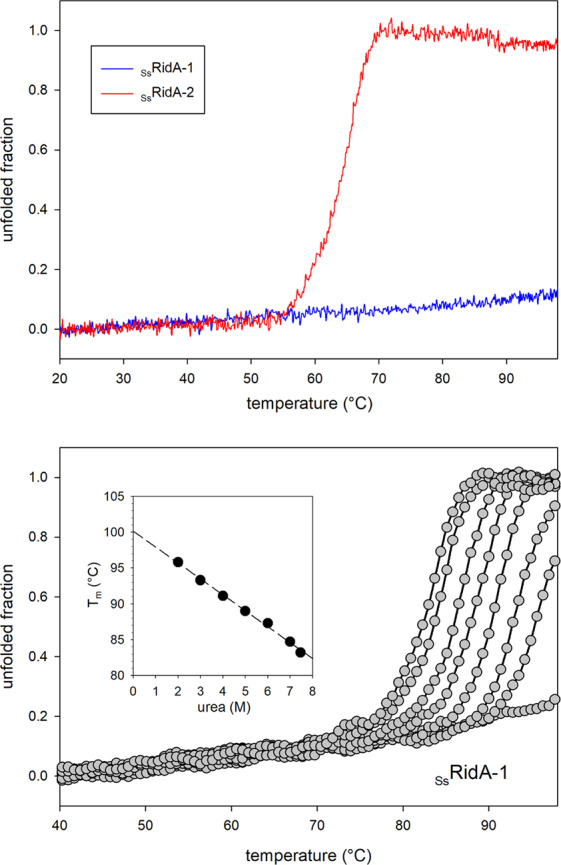
Thermal stability of _Ss_RidA-1 and _Ss_RidA-2. The fraction of unfolded protein was determined from the changes in CD ellipticity at 220 nm. *Upper panel*: temperature-dependence of the CD signal of _Ss_RidA-1 and _Ss_RidA-2 in physiological saline solution (0.9% NaCl)*. Lower panel*: temperature-dependence of the CD signal of _Ss_RidA-1 recorded in the presence of increasing concentration of urea. *Inset*: linear fitting of the calculated *T*_*m*_ values as a function of urea concentration.

The conformational stability of the *S. salar* RidA proteins was studied by monitoring the change in ellipticity at 220 nm, which reports on the α- helical content of the proteins, by varying the temperature from 20 to 98 °C. An almost flat trace was obtained for _Ss_RidA-1, indicating that the protein conformation was not affected in this temperature range (Fig. 5, *upper panel*). This behavior has been previously reported also for _Ch_RidA^25^, indicating that both goat RidA and salmon RidA-1 have a remarkable resistance to thermal unfolding. _Ss_RidA-1 thermal stability was further investigated by repeating the experiment in the presence of increasing concentrations of urea (Fig. 5, *lower panel*). The melting temperature (*T*_*m*_) in the absence of urea was extrapolated from the linear fitting of *T*_*m*_ as a function of urea concentration (*inset* of Fig. 5, *lower panel*). The calculated value of 100.2 °C is slightly lower than that calculated for _Ch_RidA (104 °C)^25^.

On the other hand, _Ss_RidA-2 showed a more canonical denaturation curve revealing a *T*_*m*_ of 65 °C (Fig. 5, *upper panel*). These results indicate that, despite the high similarity, _Ss_RidA-1 and _Ss_RidA-2 have very different conformational stability: the former is resistant to heat denaturation while the latter denatures at a temperature normally observed for mesophilic proteins^34^.

### *S. salar* RidA proteins are trimers

**Figure 6:**
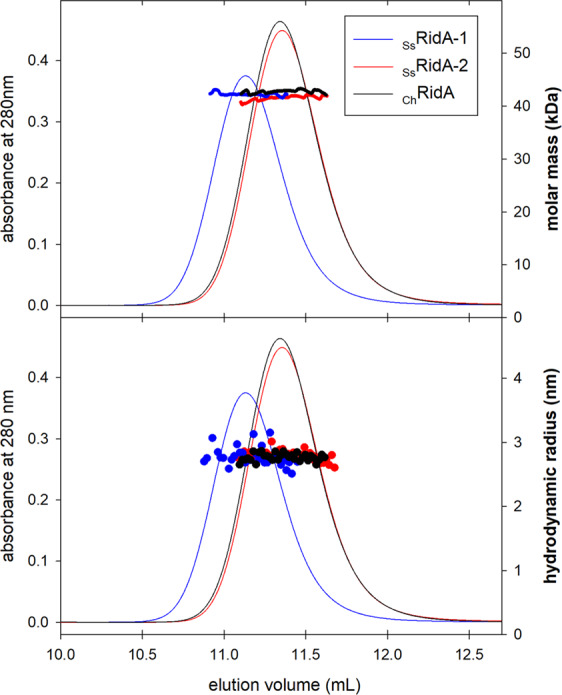
Quaternary structure of _Ss_RidA-1 and _Ss_RidA-2 and comparison with _Ch_RidA. Thin lines: SEC chromatograms at 280 nm. Thick line on the *upper panel*: molar masses calculated across the peaks by MALS. Dots in the *lower panel*: hydrodynamic radius calculated across the peaks by DLS.

CD analyses indicated that salmon RidA-2 is less stable than RidA-1. To analyze whether this could be ascribed to a different assembly in solution, the quaternary structure of the two isozymes, in comparison with _Ch_RidA, were characterized by size-exclusion chromatography (SEC) combined with multi-angle light scattering (MALS) and dynamic light scattering (DLS), (Fig. 6). The molar masses of the two paralogous *S. salar* RidA proteins in solution are similar to that of _Ch_RidA (_Ss_RidA-1: 42.2 ± 2.0 kDa; _Ss_RidA-2: 42.7 ± 2.0 kDa; _Ch_RidA: 41.5 ± 2.0 kDa). Therefore, *S. salar* RidA-1 and RidA-2 assemble into homo-trimers, as previously reported for _Ch_RidA and for other characterized orthologous proteins^25,35–37^. Moreover, the mass calculated across the protein peaks is constant suggesting that the samples are monodispersed with no evidence of dissociation equilibria affecting the trimeric association (Fig. 6, *upper panel***)**. To shed light on the cause of the lower elution volume of _Ss_RidA-1 (11.13 mL) in comparison to _Ss_RidA-2 (11.35 mL) and _Ch_RidA (11.34 mL), the hydrodynamic radius (Rh) was calculated for each protein by DLS in the same chromatographic runs. As for the molar mass, also the Rh values of the three RidA forms are similar to each other (Fig. 6, *lower panel***)** with an average value of 2.8 nm. These results rule out differences in shape or compactness of the proteins. Therefore, the different elution volume is most likely due to nonspecific electrostatic interactions between the proteins and the chromatographic matrix, being _Ss_RidA-1 the only one having a net negative charge under the experimental condition used compared to the net positive charge of _Ss_RidA-2 and _Ch_RidA (calculated pI: _Ss_RidA-1, 5.26; _Ss_RidA-2, 8.05; _Ch_RidA, 6.78; mobile phase, pH 5.8).

**Figure 7:**
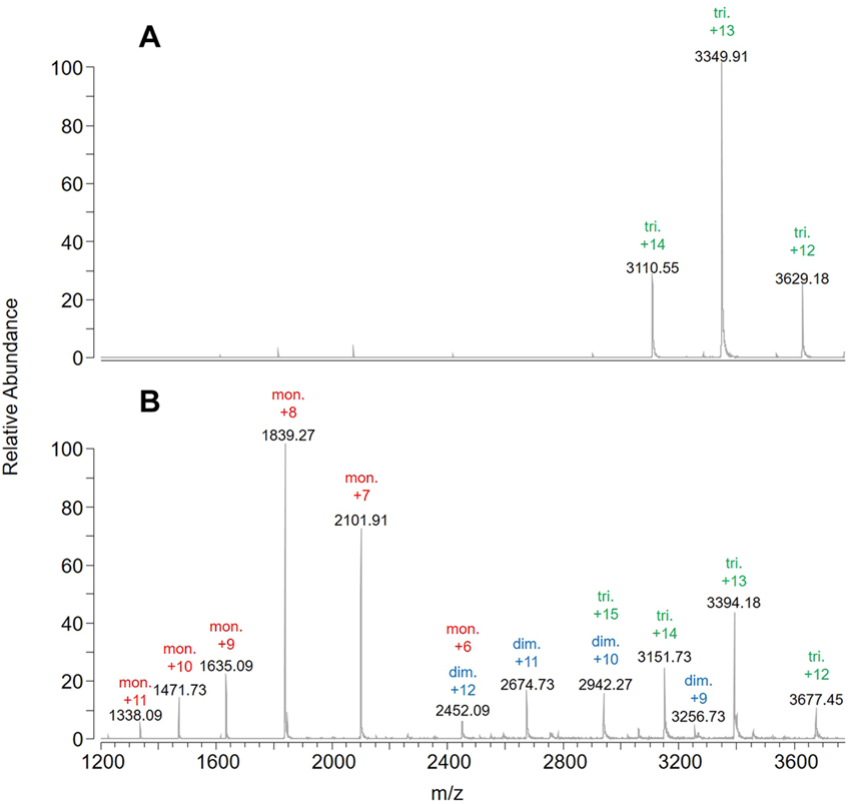
Native MS analysis. ESI mass spectra of 0.5 mg/mL _Ss_RidA-1 and 0.5 mg/mL _Ss_RidA-2 in 10 mM ammonium formate. Multiply charged ions of monomer (mon.), dimer (dim.) and trimer (tri.) of the two proteins are labeled in red, blue and green, respectively. (**A**) _Ss_RidA-1; (**B**) _Ss_RidA-2.

Further insights into _Ss_RidA-1 and _Ss_RidA-2 quaternary structure assembly and stability were provided by native MS experiments. Specifically, the _Ss_RidA-1 electrospray ionization (ESI) mass spectrum (Fig. 7A) was characterized by signals resulting from three multiply charged ions of the protein trimer (deconvoluted mass 43536 Da), whereas _Ss_RidA-2 ESI mass spectrum (Fig. 7B) was characterized by several signals resulting from multiply charged ions of protein monomer, dimer and trimer (deconvoluted mass 14707, 29412 and 43115 Da, respectively). To clarify how _Ss_RidA-2 monomer and dimer originated, the _Ss_RidA-2 sample was subjected to fractionation by ultrafiltration using 30 kDa-cutoff membranes that are suitable to separate the trimer (retentate) from the monomer (permeate). No trace of protein was detectable in permeate whereas the monomer, dimer and trimer were still detectable by native MS in the retentate yielding a spectrum superimposable to that obtained for the unfractionated sample shown in Fig. 7B, (data not shown). Taken together, the results provide evidence that _Ss_RidA-2 monomer and dimer in Fig. 7B are produced by dissociation of the _Ss_RidA-2 trimer during MS analysis. Interestingly, no trace of monomeric or dimeric forms was found under the same experimental condition for _Ss_RidA-1 (Fig. 7A), supporting the notion that _Ss_RidA-1 trimer is intrinsically more stable than the _Ss_RidA-2 one.

### _Ss_RidA-1 and _Ss_RidA-2 high resolution structures

**Figure 8:**
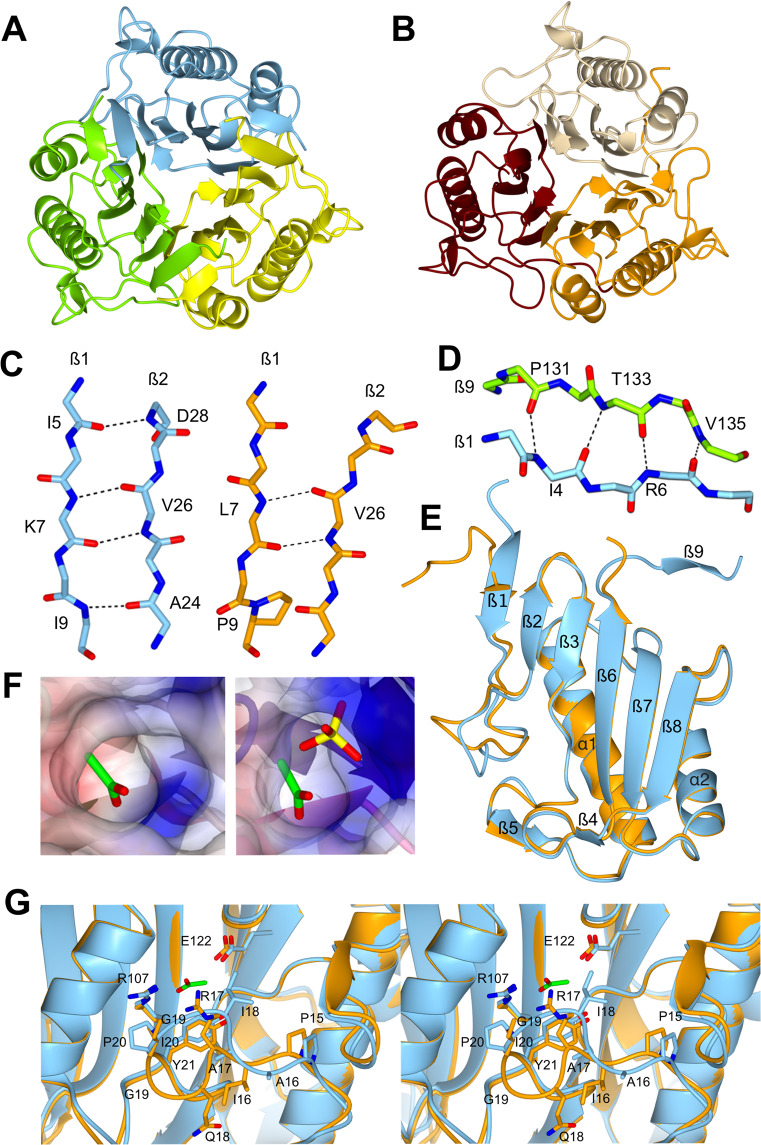
Crystal structures of _Ss_RidA-1 and _Ss_RidA-2. (**A**) Ribbon representation of the _Ss_RidA-1 trimer. (**B**) Ribbon representation of the _Ss_RidA-2 trimer. (**C**) Stick representation of β1 and β2 strands of _Ss_RidA-1 and _Ss_RidA-2 in cyan and orange, respectively. H-bonds are shown as dashed lines. In _Ss_RidA-2, the presence of Pro9 shortens β1 strand and reduces the H-bond network. (**D**) Stick representation of β1 and β9 strands of the adjacent monomer present in the _Ss_RidA-1 trimer. (**E**) Superposition of _Ss_RidA-1 and _Ss_RidA-2 monomers color-coded as in C. (**F**) Zoom on the active site cavity of _Ss_RidA-1(*left*) and _Ss_RidA-2 (*right*) shown as surface colored according to the electrostatic potential. _Ss_RidA-2 clearly displays a stronger electropositive cavity than _Ss_RidA-1. Acetate molecules (in green) present in the active site of both salmon RidA isoforms and the sulphate ion (in yellow) present in _Ss_RidA-2 are shown in sticks. (**G**) Stereo-view of the _Ss_RidA-1 and _Ss_RidA-2 active sites, color-coded as in C.

In order to better understand the structural bases of the observed differences in terms of protein stability and of enzymatic activity, the crystal structures of _Ss_RidA-1 and _Ss_RidA-2 were determined at 1.05 Å and at 1.36 Å resolutions, respectively (Table S4). As for the previously reported structures of proteins belonging to YjgF/YER057c/UK114 superfamily, the two isozymes display a trimeric assembly in agreement with the solutions studies (Fig. 8A,B)^35–38^. The _Ss_RidA-1 trimer superposes very well with the two trimers of _Ss_RidA-2 present in the asymmetric unit [pairwise root mean square deviation (r.m.s.d.) values 0.73 Å/373 Cα and 0.75 Å/375 Cα] and with the structure of _Ch_RidA (PDB: 1NQ3; r.m.s.d. 0.73 Å/398 Cα)^38^. The two _Ss_RidA-2 trimers are virtually identical (r.m.s.d. values 0.45 Å/380 Cα) and also _Ss_RidA-2 monomers are all well superposable (r.m.s.d. values in the 0.15–0.26 Å range over the whole Cα chain). Monomeric _Ss_RidA-1 and _Ss_RidA-2 share the chorismate mutase-like fold common to all the members of the YjgF/YER057c/UK114 superfamily^38^: monomers fold in a single compact domain of one six-stranded β-sheet packed against two α-helices (Fig. 8A,B,E). Thus _Ss_RidA-1 and _Ss_RidA-2 tertiary and quaternary structures are very closely related.

Nevertheless, fine structural comparisons highlighted relevant differences between _Ss_RidA-1 and _Ss_RidA-2. The sequence of strand β1 is generally poorly conserved among RidA. However, _Ss_RidA-2 is the only ortholog with a Pro residue in position 9 in place of Ile or Val in the other analysed sequences (Fig. 2 and Fig. S1). Residue 9 is part of strand β1 and a substitution of Val or Ile, which are highly compatible with a β-structure, with Pro, a β-breaker, results in a shorter and less regular β1 strand in _Ss_RidA-2 compared to _Ss_RidA-1 (Fig. 8C). Moreover, in _Ss_RidA-1, an extra intermolecular β sheet is formed between residues Ile4-Arg6 of each monomer with the C-terminal Pro131-Val135 of the neighbouring molecule in the trimer (Fig. 8D). In _Ss_RidA-2 this intermolecular two-strand β-sheet is not present and both regions are partially disordered in the structure. Such observations help to rationalise the remarkable difference in terms of protein stability between _Ss_RidA-1 and _Ss_RidA-2. Indeed, _Ss_RidA-2 lacks the intermolecular β sheet between N- and C-terminal segments present in _Ss_RidA-1 and in _Ch_RidA. This feature likely accounts for the destabilization of the trimer observed in the native MS experiments. Secondly, the shortening of β1strand by Pro9 reduces the H-bond network in the central β sheet likely decreasing the stability of the monomeric fold. Also in other systems, the insertion of Pro residues increases geometrical strain and decreases protein stability^39,40^.

RidA active site has been reported to lie at the interface between monomers^37^. In every molecule of both _Ss_RidA-1 and _Ss_RidA-2, an acetate molecule was visible in correspondence of Arg107 at the monomer-monomer interface (in green in Fig. 8F). Acetate ions are coordinated by the conserved residues Tyr21, Arg107 and Glu122, reported to be involved in the interaction with substrates in human RidA [hp14.5 protein, PDB: 1ONI^35^] and bacterial TdcF protein [PDB: 2UYN^37^]. The acetate molecules likely mimic the carboxylate group of RidA substrates. While the overall geometry of the active site is highly conserved in all the analysed RidA and also in _Ss_RidA-1, _Ss_RidA-2 displays a different conformation in the 15–20 region of the loop responsible for substrate recognition (Tyr10 - Ala24)^37^ (Fig. 8G). _Ss_RidA-2 is the only sequence that presents an Ile instead of a Pro in position 20 (Fig. 2): the conformation of this loop in _Ss_RidA-2 is possible by the greater geometrical freedom of Ile20, compared to that of Pro20, which in such conformation would fall in the non-allowed region of the Ramachandran plot. In other words, the conformation of the 15–20 loop in _Ss_RidA-2 is specific to this isozyme because of the presence of Ile20. Crucially, residues 16–18 are typically small and hydrophobic in RidA primary structures (Fig. 2), while Arg and Gln are present in position 17 and 18, respectively, in _Ss_RidA-2. This confers very different chemical properties to _Ss_RidA-2 binding pocket compared to _Ss_RidA-1. Consistently, in one of the three catalytic pockets of the SsRidA-2 trimer a sulphate ion (in yellow in Fig. 8F) is found together with the acetate molecule in close vicinity to Arg17, further stressing the strongly positive nature of _Ss_RidA-2 active site.

The different chemical properties of the 15–20 loop help to rationalise the different substrate specificities of _Ss_RidA-1 and _Ss_RidA-2. _Ss_RidA-1 is an efficient catalyst with hydrophobic IA and possesses a mainly hydrophobic 15–20 loop. On the other hand, _Ss_RidA-2 has a positive charge in position 17 and, under our experimental conditions, showed a strong preference for the IA derived from L-Glu, while the lack of activity on the IA derived from L-Asp cannot be explained. These considerations suggest that the 15–20 loop is crucial in determining the distinct substrate specificity of RidA enzymes.

## Discussion

RidA activity is ubiquitous and the conservation from bacteria to mammals underlies its importance in preventing metabolic stress^1,25^. Unlike most vertebrates, salmon and other teleosts contain two paralogous copies of the mammalian *ridA* gene. The high sequence similarity between _Ss_RidA-1 and _Ss_RidA-2 and the partial conservation in synteny between the underlying loci are largely consistent with their origin from a WGD event. However, considerations concerning sequence similarity, substrate specificity, conformational stability and structural features indicate that _Ss_RidA-1 is the *bona fide* functional ortholog of the vertebrate RidA. The presence of RidA is consistent with the presence of L-serine dehydratase in salmon as annotated in Salmobase database.

The striking different catalytic efficiencies with different IA substrates between the salmon RidAs suggest that these enzymes could play complementary functions in bony fish. Like the goat enzyme, _Ss_RidA-1 could play the predominant function in the deamination of IA derived from nonpolar amino acids (L-Leu, L-Ala and L-Met) and be less active on IA derived from aromatic amino acids, substrates never tested before, and inactive on those derived from charged residues. In contrast, _Ss_RidA-2 has a low catalytic efficiency with nonpolar substrates, but it has a high activity on the imino acid deriving from L-Glu indicating an opposite substrate preference with respect to _Ss_RidA-1 and goat RidA. _Ss_RidA-2 also displayed a relatively higher catalytic efficiency than other RidA on IA from aromatic amino acids, except the L-Trp derivative on which _Ch_RidA is more active. These findings are important as they suggest that proteins of the RidA family could also be involved in the metabolism of L-Glu and aromatic amino acids or their derivatives among which are neurotransmitters and their precursors, i.e. L-Glu, 5-hydroxy tryptamine and L-DOPA. In this respect, it is worthwhile noting that in mammals, *ridA* gene expression is high in the liver, low in the kidney cortex and also in some areas of the brain except the cerebellum (http://genome.ucsc.edu). Finally, the two salmon RidA enzymes are endowed with activity that probably differs due to a specialization of the duplicated *ridA* gene that has been retained since, as suggested by genetic evidence, diploidy in salmon is not yet fully re-established^29^. However, hints to potential novel roles of RidA, other than protection from metabolic damage by 2AA derived from serine dehydratase and cysteine desulfhydrase, can emerge from our study.

## Methods

### *In silico* analyses

Complete proteomes were retrieved from NCBI GenBank for *S. salar* and *C. hircus* and from the UCSC Genome browser for all the other species. Details concerning assembly versions and genomic annotations are reported in Table S5. All against all BLASTP^41^ were performed using the BLOSUM80 matrix^42^ and accepting only best reciprocal hits with an e-value ≤ 1e^−5^, which covered at least 50% of the protein length, and where “second-best” hits producing bit scores <90% of that associated with the best match. Putative orthologs were identified as best reciprocal BLAST hits by means of a custom Perl script. Synteny analyses were performed by cross-referencing putative orthologous proteins with the corresponding annotation in gtf format in order to derive genomic coordinates and the underlying gene structures, again by means of a custom Perl script.

The sequences of RidA-like salmon proteins were retrieved by performing a BLASTP similarity search, applying the default parameters, in the salmon proteome using the human RidA as a query. A second similarity search using BLASTN or BLASTP in the *S. salar* database at Salmobase 2.0 (https://salmobase.org/) was performed to retrieve the corresponding cDNA sequences and genomic coordinates and sequences. The multiple alignment was done with Clustal Omega https://www.ebi.ac.uk/Tools/msa/clustalo/ and Box Shade https://embnet.vital-it.ch/software/BOX_doc.html using default parameters. The WebLogo was created using the software at https://weblogo.berkeley.edu.

### Bacterial strains, media and chemicals

Bacterial strains used in this work are listed in Table S1 and were grown in Luria-Bertani medium [0.5% (w/v) yeast extract, 1% (w/v) peptone, 0.1% (w/v) glucose, 0.5% (w/v) NaCl] supplemented with 100 µg/mL ampicillin and 20 µg/mL chloramphenicol.

In the complementation study, derivatives of *S. enterica* serovar Typhimurium LT2 were used. Minimal medium was no-carbon E supplemented with 20 mM glycerol as the sole carbon source, 1 mM MgSO_4_^43^, and trace minerals^44^. Difco nutrient broth (NB) (8 g/L) supplemented with 5 g/L NaCl was used as rich medium for *S. enterica*. Ampicillin was used in rich (150 μg/mL) and minimal (7.5 μg/mL) medium for plasmid maintenance, when appropriate.

### Plasmid construction and recombinant protein production

Recombinant plasmids for the expression of _Ss_RidA-1and _Ss_RidA-2 in *E.coli* were obtained by GENEWIZ GmbH (Leipzig, Germany) by cloning in the pET-15b vector double-digested with *Nde*I and *Xho*I, the synthesized DNA of the CDS. The fusion proteins carried at the N-terminus, the sequence MGSSHHHHHHSSGLVPR/GSH, comprising a 6xHis-tag and the cleavage site for thrombin (/) inside the thrombin recognition site (underlined). The proteins were expressed from *E. coli s*trains harbouring the recombinant plasmids (Table S1) and purified as detailed in^25^. This procedure yielded about 200 mg and 66 mg of homogeneous _Ss_RidA-1 and _Ss_RidA-2 from 3 L cultures, respectively.

For complementation tests, the CDS of _Ss_RidA-1 (UniprotKB A0A1S3KNQ3) and _Ss_RidA-2 (UniprotKB C0H8l4) were codon optimized for expression in *S. enterica* LT2 using the online application OPTIMIZER (http://genomes.urv.es/OPTIMIZER/)^45,46^ and synthesized by GenScript (Piscataway, NJ), (see Fig. S4). Synthesized sequences contained *Bsp*QI restriction sites flanking the gene of interest. Genes were excised and transferred into a *Bsp*QI-modified pBAD24 vector (pCV1)^47^ for downstream use in complementation of an *S. enterica ridA* mutant by *Bsp*QI restriction cloning^48^. The resulting plasmids encoded _Ss_RidA-1 (pDM1616) and _Ss_RidA-2 (pDM1617) under the control of the arabinose-inducible *P*_*BAD*_ promoter. pCV1-*ridA* (pDM1439), which harbored the *ridA* gene from *S. enterica* LT2, had been constructed previously^23^.

### Assay of RidA imine deaminase activity

The assay is based on the use of L-amino acid oxidases to generate IAs from appropriate amino acids^8,9,25^. The spectrophotometric assay measures the initial rate of formation of semicarbazone species derived from the spontaneous reaction of the IA with semicarbazide in the absence or presence of varying RidA concentrations. By accelerating the hydrolysis of IA to the corresponding ketoacid and ammonia, RidA subtracts IA to the reaction with semicarbazide leading to a decrease of the measured initial velocity of the reaction.

The reaction mixtures were set-up in quartz microcuvettes (10 mm light path) and were composed of 5 mM L-amino acid, except for the L-aromatic amino acids the concentration of which was 0.5 mM, 10 mM neutralized semicarbazide-HCl, catalase, 6.6 μg/ml, snake venom L-amino acid oxidase [Sigma cat #A9378, 8–50 µg/mL), or L-glutamate oxidase (Sigma cat# G-5921) or L-aspartate oxidase (a kind gift of Prof. Armando Negri and Gabriella Tedeschi, University of Milan) and different amounts of RidA enzymes in 50 mM sodium pyrophosphate buffer, pH 8.7^25^. The amount of the L-amino acid oxidase was adjusted in order to have similar rates of formation of IA with the different L-amino acids (~ 0.2–0.3 ΔA_248_/min). The reaction was started by the addition of the L-amino acid and monitored as described in^25^. The time-course of formation of the semicarbazone species was monitored as the increase of absorbance at 248 nm. The initial reaction velocity measured in the presence of a given concentration of RidA (*v*_RidA_) was then expressed as percent of the initial velocity measured in the absence of RidA (*v*_o_). The resulting residual velocity (*v*) values were then fitted with equation (1) using the GraFit 4.0 software (Erythacus Software Ltd) to calculate the K_50_ value and the associated error^25^.

In Equation (1), *v* equals (*v*_RidA_/*v*_o_ × 100), K_50_ is the RidA concentration that halves the initial rate of semicarbazone formation.

1 $$\upsilon =\frac{100}{1+\frac{[{\rm{RidA}}]}{{K}_{50}}}$$

As detailed in^25^, 100/K_50_ is a measure of the catalytic efficiency of the RidA with the imino acid produced by the L-amino acid oxidase in the coupled assay. The higher the K_50_ value, the lower is the efficiency of the enzyme in catalysing the reaction with the specific substrate. When the activity of RidA was very low, the residual activity values were fitted to a straight line. In this case the absolute value of the slope corresponds to 100/K_50_.

### Complementation of an *S. enterica ridA* mutant

Overnight cultures of the *S. enterica ridA* mutant strains harbouring pCV1 (vector only), pDM1439 (*S. enterica ridA*), pDM1616 (_Ss_RidA-1), and pDM1617 (_Ss_RidA-2) (Table S1) were grown in biological triplicate at 37 °C in NB medium containing ampicillin and were used to inoculate (1:100) 200 μl of minimal glycerol growth medium containing 5 mM L-serine, 0.2% (wt/vol) L-arabinose, and ampicillin. 96-well plates were incubated at 37 °C in a microplate reader (model ELx808; BioTek Instruments) and shaken at low speed. Growth was monitored for 24 h by monitoring the OD_650_ changes. Results were plotted in log_10_ format using GraphPad Prism 7.0d and represent the averages and standard error of the means from the replicates.

### Analytical size exclusion chromatography - light scattering

SEC combined with MALS and DLS detection were performed in a HPLC system composed by a Waters 515 HPLC Pump, a Waters 2487 Dual λ Absorbance detector (Waters, Sesto San Giovanni, Italy), a Wyatt Dawn Heleos MALS equipped with a DLS and a Wyatt Optila T-rEX differential refractive index detector (Wyatt technology, Santa Barbara, Ca).

200 μL of 1 mg/mL protein samples were chromatographed on a Superdex 75 column (10/300 GL, GE Healthcare, Milan, Italy) equilibrated and eluted with physiological saline solution (0.9% w/v NaCl) at a flow rate of 0.5 mL/min. Molar masses and Rh were calculated by means of the Astra V software vs. 5.3.4.20 (Wyatt), using a dn/dc value of 0.185.

### Circular dichroism and MS analyses

CD spectra and thermal denaturation experiments were carried out as previously described^25^. Apparent *T*_*m*_ values were determined as the maximum of the first derivative of the unfolding profiles. With _Ss_RidA-1, heat denaturation was also studied in the presence of urea concentrations ranging from 2 to 7.5 M.

MS experiments were performed according to a previously described protocol^25^. Buffer exchange and fractionation for native MS experiments were performed by using Amicon Ultra-0.5 mL centrifugal concentrators. Devices equipped with 3 kDa-cutoff membranes were used to exchange the protein storage solution with aqueous 10 mM ammonium formate; filters equipped with 30 kDa cutoff membranes were used to separate the protein species with mass <30 kDa (permeate) from assemblies with mass >30 kDa (retentate).

### Protein crystallization

Purified _*Ss*_RidA-1 and _*Ss*_RidA-2 were concentrated up to 7.5 mg/mL in 154 mM NaCl solution and subjected to crystallization trials using an Oryx4 crystallization robot (Douglas Instruments). Crystallization screening experiments were performed in sitting drop technique by mixing the protein sample with crystallization reservoir solution at three different protein: precipitant ratios (30%:70%, 50%:50%, 70%:30% in droplets) in a final volume of 0.6 µl, equilibrated over 100 µl of the reservoir solution. Crystallization plates were incubated at 20 °C.

_Ss_RidA-1 single and sizable crystals suitable for X-ray diffraction experiments grew overnight from PACT condition A7 (0.2 M sodium chloride, 0.1 M sodium acetate, pH 5.0, 20% PEG 6000), they were cryo-protected adding 25% glycerol and flash frozen in liquid nitrogen. _Ss_RidA-2 microcrystal hits were observed at 20 °C in the condition B8 of Crystal Screen 1 (Hampton Research) containing 0.2 M ammonium sulfate, 0.1 M Na acetate, pH 4.6, 25% PEG 4000. The condition was optimized to 0.1 M ammonium sulfate, 0.1 M sodium acetate trihydrate, 25% w/v PEG 4000, by manual crystallization. _Ss_RidA-2 crystals were mounted in cryo-loops and flash frozen without additional cryo-protectant.

### X-ray data collection and structure determination

X-ray diffraction data were collected at cryogenic temperature (100 K) at the Diamond Light Source (DLS, Didcot, United Kingdom) on beamlines I03 and I04, both equipped with a Dectris Eiger2 XE 16 M detector. _Ss_RidA-1 reflections were integrated using XDS^49^ and scaled with Scala^50^ from the CCP4 suite^51^. Diffraction data from _Ss_RidA-2 were processed with the autoPROC software^52^. The dataset revealed anisotropic diffraction. Therefore, it was submitted to the STARANISO program, which performs an anisotropic cut-off of merged intensity data, a Bayesian estimation of structure amplitudes and applies an anisotropic correction to the data^53^.

Both _Ss_RidA-1 and _Ss_RidA-2 structures were determined by molecular replacement by MOLREP^54^ using as search model the coordinates of the human hp14.5 protein [PDB ID 1ONI^35^], showing 71% and 67% sequence identity with _Ss_RidA-1 and _Ss_RidA-2, respectively. Specifically, the monomeric template was used for _Ss_RidA-1 and a whole trimeric ensemble for _Ss_RidA-2.

Initial molecular replacement solutions were subjected to subsequent cycles of manual building in Coot^55^ and refinement with phenix.refine^56^. Ligand occupancy was estimated using a criterion based on keeping the atomic displacement parameters of each molecule comparable with those of the surrounding residues (in fully occupied sites). Water molecules were added with the program ARP/wARP^57^ and visually inspected with Coot^55^. For _Ss_RidA-1 structure, in the last cycle of refinement, hydrogen atoms were added in calculated positions, contributing to the Fc calculation. Models were inspected and validated using Molprobity^58^; processing and refinement statistics are reported in Table S4. Structural images were generated using CCP4mg^59^.

## Supplementary information

Supplementary information.

## Data Availability

The structures presented in this paper have all been deposited in the Protein DataBank (PDB database: https://www.rcsb.org/) with the following codes: 6TCC for _Ss_RidA-1 and 6TCD for _Ss_RidA-2. All the materials, protocols and strains generated by this study are available upon request.
