# Supplementary material for: Two novel fish paralogs provide insights into the Rid family of imine deaminases active in pre-empting enamine/imine metabolic damage
Source: Sci Rep. 2020 Jun 23;10:10135. doi: 10.1038/s41598-020-66663-w (PMC7311433; doi:10.1038/s41598-020-66663-w)
Supplement: Supplementary file 1 — Supplementary information. [file 41598_2020_66663_MOESM1_ESM.pdf]

Supplementary information

Two novel fish paralogs provide insights into the Rid family of imine deaminases active in pre-empting enamine/imine metabolic damage

Stefania Digiovanni[1,#], Cristina Visentin[1], Genny Degani[1], Alberto Barbiroli[2], Matteo Chiara[1], Luca Regazzoni[3], Flavio Di Pisa[1], Andrew J. Borchert[4,‡], Diana M. Downs[4], Stefano Ricagno[1], Maria Antonietta Vanoni[1,§] and Laura Popolo[1,*]

[1]Department of Biosciences, [2] Department of Food, Environmental and Nutritional Sciences and [3] Department of Pharmaceutical Sciences, University of Milan, Italy; [4]Department of Microbiology, University of Georgia, Athens, GA

[§]Co-corresponding author

*Corresponding author

Figure S1

Table S1

Figure S2

Table S2

Figure S3

Table S3

Figure S4

Table S4

Table S5

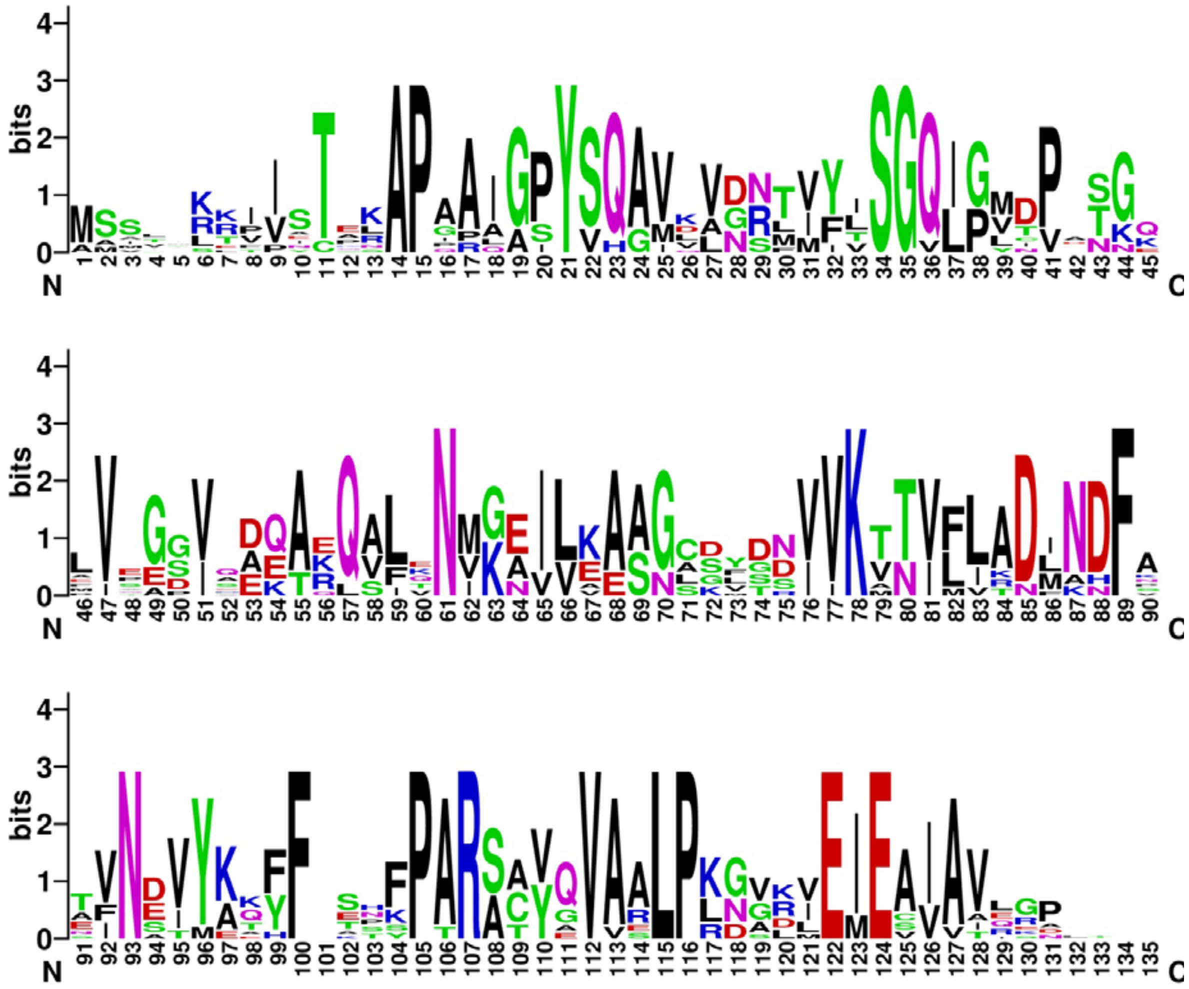

Figure S1

WebLogo of the RidA sequences of the multiple alignment shown in **Fig. 2**. Amino acids are color coded according to the WebLogo standard settings as follows: G, S, T, Y, C, Q, N are green, K, R, H are blue, D, E red and A, V, L, I, P, W, F, M are black. To align the essential Arg105 of prokaryotes with the essential Arg107 of eukaryotes, three not conserved amino acids at position104-106 (HQA in *E. coli* and HNA in *S. typhimurium*) were removed to generate the WebLogo, which requires sequences of identical length. The numbering is that of eukaryotic RidA.

Table S1. Bacterial strains and plasmids

	Genotype	Plasmid	Notes	Source
E. coli *Rosetta (DE3)*				Lab stock
SD1	pRARE (Cm^R) pET15b-$_{Ss}$RidA-1 (Amp^R)	pSD1	Production of $_{Ss}$RidA-1	This work
SD2	pRARE (Cm^R) pET15b-$_{Ss}$RidA-2 (Amp^R)	pSD2	Production of $_{Ss}$RidA-2	This work
GD1	pRARE (Cm^R) pET15b-$_{Ch}$RidA-2 (Amp^R)	pGD1	Production of $_{Ch}$RidA	(25)
S. enterica				
DM14829	*ridA1*::*Tn10d* (Tc)	None		Lab stock
DM16362	*ridA1*::*Tn10d* (Tc)	pCV1	*Bsp*QI-modified pBAD24	(47)
DM16360	*ridA1*::*Tn10d* (Tc)	pDM1439	pCV1-*ridA*	(23)
DM16978	*ridA1*::*Tn10d* (Tc)	pDM1616	pCV1-$_{Ss}$RidA-1	This work
DM16979	*ridA1*::*Tn10d* (Tc)	pDM1617	pCV1-$_{Ss}$RidA-2	This work

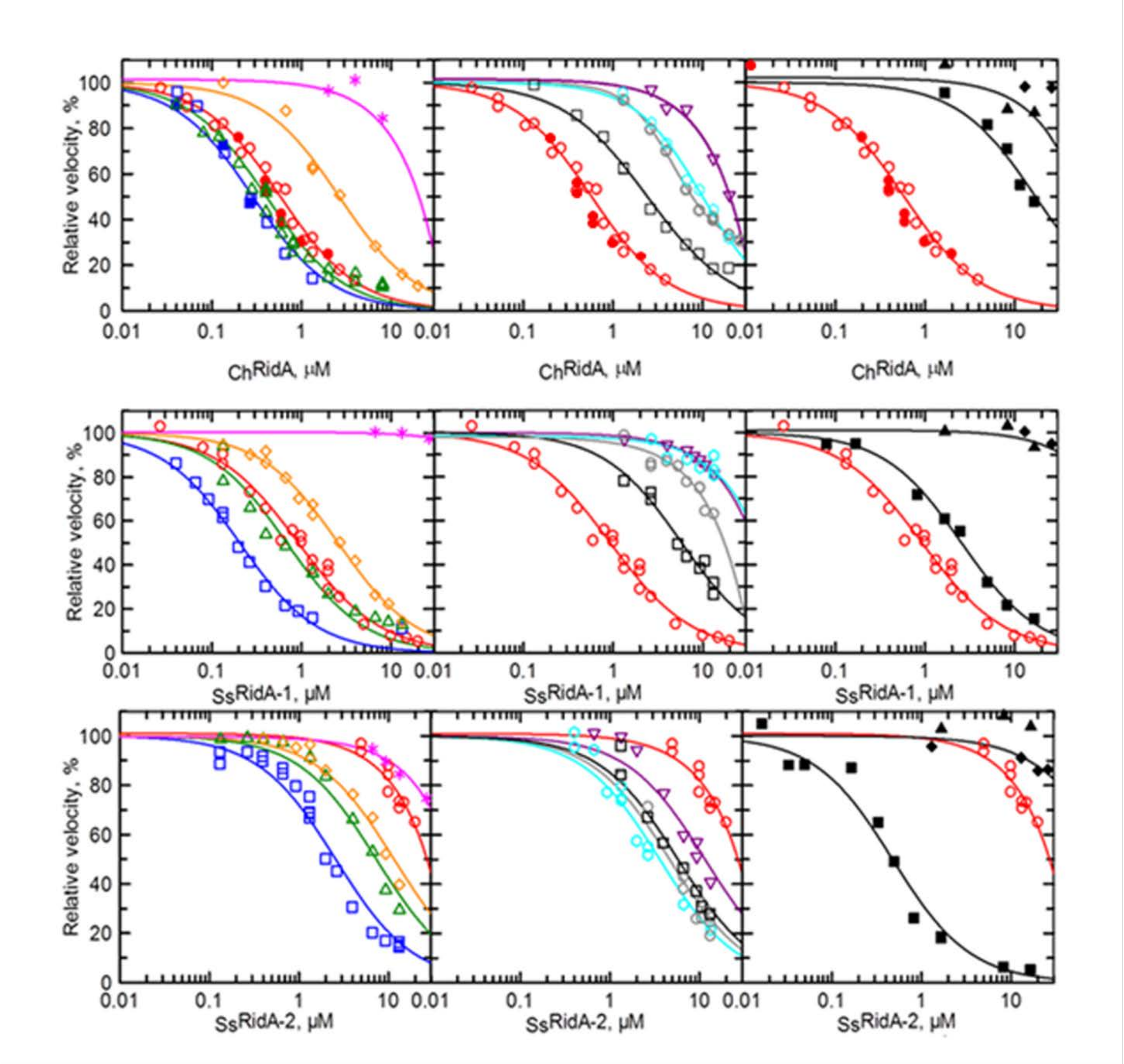

Figure S2. Specificity of $_{Ch}$RidA, $_{Ss}$RidA-1 and $_{Ss}$RidA-2. *Left panels*: L-Ala (open squares, blue), L-Leu (open circles, red), L-Met (open triangles, green), L-Gln (open diamonds, orange), L-His (stars, fuchsia). *Middle panels*: L-Leu (open circles, red), L-Phe (inverted triangles, purple), L-Tyr (open hexagons, light blue), L-Trp (open squares, black), L-DOPA (open circles, grey). *Right panels*: L-Leu (open circles, red), L-Glu (closed squares, black), L-Asp (closed diamonds, black). L-Leu is reported in all panels as an internal reference. For the $_{Ch}$RidA, D-Leu (closed circles, red), which was reported in the previous study in which also D-amino acid oxidase was used (25), is also shown as an internal reference. The experimental points represent the initial velocity of semicarbazone production measured in the presence of RidA (v_{RidA} expressed as percent of that measured in the absence of RidA (v_o), i.e. [v_{RidA}/v_o x 100]. The amount of L-amino acid oxidases used to generate the imino acid substrate of RidA from different amino acids was adjusted in order to obtain similar v_o values in the range 0.2-03 ΔA_{248}/min. Data were fitted to Equation 1, obtaining the values of the concentration of RidA proteins that halves the velocity of semicarbazone formation (K_{50}), from which the 100/K_{50} values summarized in Table S2 were obtained. The data obtained with L-Phe were fitted with a straight line. In this case 100/K_{50} is the absolute value of the slope of the line.

Table S2. Specificity of salmon RidA proteins compared to goat RidA

	$100/K_{50}$[a], μM^{-1}		
Amino acid[a]	$_{Ch}$RidA	$_{Ss}$RidA-1	$_{Ss}$RidA-2
L-Leu	172.0±9.4	112 ± 9	1.90±0.15 [b]
L-Ala	348±24	489±23	38.9±2.2
L-Met	245±16	146±14	13.6±1.1
L-Gln	37.9±2.7	39.0±1.3	8.7±0.6
L-His	1.9±1 [b]	NA	0.98±0.1 [c]
L-Tyr	9.6±0.6	1.2±0.2 [b]	27.7±2.2
L-Phe	2.50±0.16 [b]	1.4±0.2 [b]	8.7±0.9
L-DOPA	10.6±0.5	2.8±0.3 [b]	21.6±1.2
L-Trp	41±2.2	17.2±0.9	17.4±1.5
L-Arg	1.0±0.5 [d]	ND [c]	ND
L-Asp	ND	ND	ND
L-Glu	5.9±0.5	40.4±3.3	210±25

[a] The values were obtained by fitting three or more independent measurements of reaction velocity *v* with Equation (1) ± standard deviation (see "Assay of RidA imine deaminase activity" under Methods).

[b]The amino acid concentration was 5 mM except for L-aromatic amino acids (0.5 mM) but the amount of the L-amino acid oxidase was adjusted to obtain similar initial velocities of imino acid formation starting from the different amino acids.

[c]Data were fitted with a straight line in which the absolute value of the slope corresponds to $100/K_{50}$

[d]No detectable RidA activity up to 20 μM RidA.

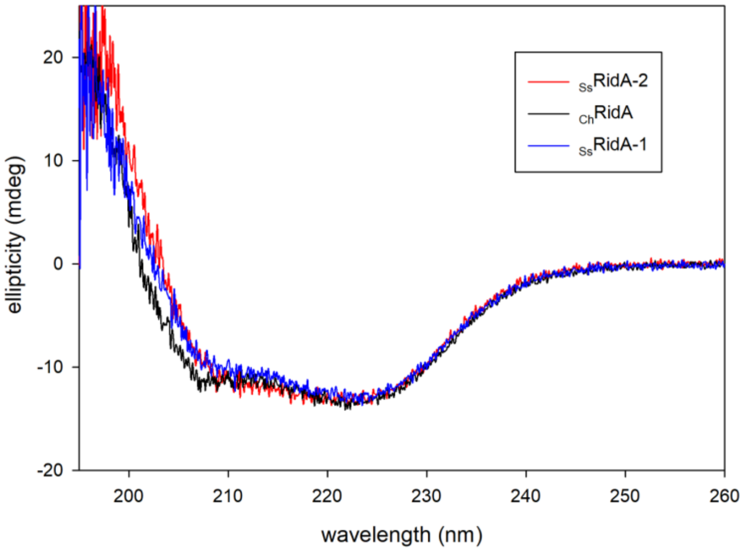

Figure S3. Far-UV CD spectra of the indicated proteins. Spectra were acquired at 20 °C in 0.1 cm path length cuvette, at 0.2 mg/mL protein concentration.

Table S3. Predicted secondary structure content of salmon and goat Rid proteins

	Predicted secondary structure content (%)		
	$_{Ss}$RidA-1	$_{Ss}$RidA-2	$_{Ch}$RidA
Helix	17.1	18.8	17.2
Antiparallel	23.2	22.4	21.9
Parallel	5.8	6.0	5.6
Beta-Turn	17.6	16.6	18.5
Random Coil	33.5	32.9	33.8

Estimate of the secondary structure composition was obtained by running CDNN CD deconvolution software, version 2.1 (Copyright © Gerald Böhm, Institut für Biotechnologie, Martin-Luther Universität Halle-Wittenberg) on spectra reported in **Fig. S3**, in the 200-260 nm region.

A

Type	Sequences	CAI	ENc	%GC	%AT
Query	ATGTCTTCGATCATCAGGAAGATAATTAACACCAGTAAAGCGCCAGCAGCTATCGGGCCG<br>TACAGCCAGGCGGTGGTGGTGGACAGGACCATGTACGTGTCAGGCCAGCTGGGGATGGAC<br>CCTGCCTCTGGTCAGCTGGTGGAAGGAGGAGTCCAGGCTCAGACCAAACAGGCTCTGGTG<br>AACATGGGGGAGATCCTGAAAGAAGCAGGGTGTGGATATGACAGTGTCGTGAAAACTACG<br>GTTCTTTTGGCTGACATGAATGACTTCGCCAGTGTAAATGACGTCTATAAAACATTTTTC<br>AGCAGTAGCTTCCCAGCGAGGGCTGCCTACCAGGTCGCTGCTCTGCCCAGGGGTGGGCTT<br>GTAGAGATTGAAGCTGTGGCTGTTCTAGGCCCTCTGACTGAGGTCTCTTGA	0.404	47	53.8	46.2
Optimizcd	ATGTCCTCCATCATCCGTAAAATCATCAACACCTCCAAAGCGCCGGCGGCGATCGGCCCG<br>TACTCCCAGGCGGTGGTGGTGGACCGTACCATGTACGTGTCCGGCCAGCTGGGCATGGAC<br>CCGGCGTCCGGCCAGCTGGTGGAAGGCGGCGTGCAGGCGCAGACCAAACAGGCGCTGGTG<br>AACATGGGCGAAATCCTGAAAGAAGCGGGCTGCGGCTACGACTCCGTGGTGAAAACCACC<br>GTGCTGCTGGCGGACATGAACGACTTCGCGTCCGTGAACGACGTGTACAAAACCTTCTTC<br>TCCTCCTCCTTCCCGGCGCGTGCGGCGTACCAGGTGGCGGCGCTGCCGCGTGGCGGCCTG<br>GTGGAAATCGAAGCGGTGGCGGTGCTGGGCCCGCTGACCGAAGTGTCCTGA	1.000	20	64.5	35.5

B

Type	Sequences	CAI	ENc	%GC	%AT
Query	ATGGCTGCTGTTCAGAAACTCTTTCCTTATACTCCTAGAGCACCTATAAGGCAGGGGATT<br>TACAGCCAGGCGGTGGTGGTGGATCGGACGATGTACATCTCCGGCCAGCTGGGGCTGGAC<br>GTGGCCTCAGGGAAGCTGGTGGAGGGAGGGGTACAGGCTCAGGCCAGACAGGCTCTGGTC<br>AATATGGGAGAGATCCTGAAAGCAGCTGGATGTGGTTATGACAATGTCGTCAAGACAACC<br>GTGCTGTTGGCAGACATGAATGACTTTGTCAATGTCAACGATGTTTATAAGACATTTTTC<br>AGCAAAAACTTCCCTGCCAGAGCTGCCTACCAGGTTGTTGCCCTCCCCAGAGGTGGCCTG<br>GTGGAGATCGAGGCTGTGGCTGTTCTGGGACCCATCTCTGAGTCCTGA	0.379	47	53.7	46.3
Optimizcd	ATGGCGGCGGTGCAGAAACTGTTCCCGTACACCCCGCGTGCGCCGATCCGTCAGGGCATC<br>TACTCCCAGGCGGTGGTGGTGGACCGTACCATGTACATCTCCGGCCAGCTGGGCCTGGAC<br>GTGGCGTCCGGCAAACTGGTGGAAGGCGGCGTGCAGGCGCAGGCGCGTCAGGCGCTGGTG<br>AACATGGGCGAAATCCTGAAAGCGGCGGGCTGCGGCTACGACAACGTGGTGAAAACCACC<br>GTGCTGCTGGCGGACATGAACGACTTCGTGAACGTGAACGACGTGTACAAAACCTTCTTC<br>TCCAAAAACTTCCCGGCGCGTGCGGCGTACCAGGTGGTGGCGCTGCCGCGTGGCGGCCTG<br>GTGGAAATCGAAGCGGTGGCGGTGCTGGGCCCGATCTCCGAATCCTGA	1.000	20	64.2	35.8

Figure S4. Codon optimized sequences used for the synthesis of (A) *ridA-1* and (B) *ridA-2* salmon gene sequences by GenScript (Piscataway, NJ), as determined using the online application OPTIMIZER (http://genomes.urv.es/OPTIMIZER/). The *Salmonella enterica* subsp. *enterica* serovar Typhimurium str. LT2 was denoted as the reference, the codon usage of the most highly expressed genes (HEG) was selected, and the one AA-one codon function was employed for making changes, which substitutes every amino acid codon with the most frequent codon in *S. enterica* LT2 HEG usage table. Sequence inputs and outputs are provided along with their corresponding codon adaptation index (CAI), effective number of codons (ENc) value, % GC content of the sequence, and %AT content of the sequence. CAI (0-1) measures the similarity between codon usage of the gene and reference, where 1 represents perfect correlation, and ENc (20-61) measures codon usage bias where 20 means that only 20 of the possible 61 codons were used.

Table S4. Data collection and refinement statistics

	$_{Ss}$RidA-1 (PDB code 6TCC)	$_{Ss}$RidA-2 (PDB code 6TCD)
**Crystal**		
Space group	H 32	P $2_1 2_1 2$
Cell dimensions *a*, *b*, *c* (Å)	52.19, 52.19, 242.56	100.89, 146.57, 53.68
**Data collection**		
Beamline	DLS I03	DLS I04
Wavelength (Å)	0.6500	0.9795
Resolution (Å)	42.35-1.05 (1.11-1.05)	83.10-1.36(1.47-1.36)
Total reflections	1169599 (164151)	1505301 (58528)
Unique reflections	60156 (8683)	114968(5748)
R_{merge}	0.057 (1.174)	0.079 (1.435)
$R_{meas,}$	0.059 (1.206)	0.082 (1.513)
$I/\sigma(I)$	21.1 (2.5)	16.5 (1.5)
$CC_{1/2}$	1.000 (0.860)	1.000 (0.628)
Completeness (%)	100.0 (100.0)	95.4 (67.2)
Redundancy	19.4 (18.9)	13.1 (10.2)
Wilson B-factor (Å)	11.7	15.1
**Refinement**		
Resolution (Å)	33.07-1.05	43.97-1.36
No. reflections	60133	114898
R_{work} / R_{free}	14.5/16.3	15.9/18.4
No. atoms		
Protein	1982	5830
Ligands	31	43
Water	94	809
B factors		
Protein	19.7	19.5
Ligands	52.6	28.3
Water	24.8	29.9
R.m.s. deviations		
Bond lengths (Å)	0.008	0.006
Bond angles (°)	1.061	0.800
Clash scores	1.95	1.52
Ramachandran		
Favored (%)	98.5	98.2
Allowed (%)	1.5	1.8

[a] Values in parentheses are for highest-resolution shell.

Table S5

Species	Genome assembly	Resource accessed	Annotation source	Link
H. sapiens	GRCh38/hg38	UCSC gb	Ncbi Refseq	https://hgdownload.soe.ucsc.edu/goldenPath/hg38/bigZips/genes/hg38.ncbiRefSeq.gtf.gz
C. hircus	CHIR_1.0	NCBI	Ncbi Refseq	https://www.ncbi.nlm.nih.gov/genome/annotation_euk/Capra_hircus/102/
G. gallus	galGal6	UCSC gb	Ncbi Refseq	https://hgdownload.soe.ucsc.edu/goldenPath/galGal6/bigZips/genes/galGal6.ncbiRefSeq.gtf.gz
X. laevis	xenLae2	UCSC gb	Ncbi Refseq	https://hgdownload.soe.ucsc.edu/goldenPath/xenLae2/bigZips/genes/xenLae2.ncbiRefSeq.gtf.gz
O. latypes	oryLat2	UCSC gb	Ncbi Refseq	https://hgdownload.soe.ucsc.edu/goldenPath/oryLat2/bigZips/genes/oryLat2.refGene.gtf.gz
G. mohrua	gadMor1	UCSC gb	Ensembl Gencode	https://hgdownload.soe.ucsc.edu/goldenPath/gadMor1/bigZips/genes/gadMor1.ensGene.gtf.gz
S. salar	ICSASG_v2	NCBI	Ncbi Refseq	https://www.ncbi.nlm.nih.gov/genome/annotation_euk/Salmo_salar/100/
C. milii	calMil1	UCSC gb	Ncbi Refseq	https://hgdownload.soe.ucsc.edu/goldenPath/calMil1/bigZips/genes/calMil1.ncbiRefSeq.gtf.gz

gb: genome browswer
